# Three-month antibody persistence of a bivalent Omicron-containing booster vaccine against COVID-19

**DOI:** 10.1038/s41467-023-38892-w

**Published:** 2023-08-23

**Authors:** Spyros Chalkias, Charles Harper, Keith Vrbicky, Stephen R. Walsh, Brandon Essink, Adam Brosz, Nichole McGhee, Joanne E. Tomassini, Xing Chen, Ying Chang, Andrea Sutherland, David C. Montefiori, Bethany Girard, Darin K. Edwards, Jing Feng, Honghong Zhou, Lindsey R. Baden, Jacqueline M. Miller, Rituparna Das

**Affiliations:** 1 Moderna, Inc., Cambridge, MA USA; 2 Meridian Clinical Research, Norfolk, NE USA; 3 Brigham and Women’s Hospital, Boston, MA USA; 4 Meridian Clinical Research, Omaha, NE USA; 5 Meridian Clinical Research, Grand Island, NE USA; 6 Department of Surgery and Duke Human Vaccine Institute, Durham, NC USA

**Keywords:** Vaccines, SARS-CoV-2

## Abstract

We previously presented day 29 interim safety and immunogenicity results from a phase 2/3 study (NCT04927065) comparing the Omicron-BA.1-containing bivalent vaccine mRNA-1273.214 (50-µg) to the 50-µg mRNA-1273 booster in adults who previously received the mRNA-1273 primary series (100-µg) and mRNA-1273 first booster (50-µg) dose. Primary endpoints were safety, non-inferiority of the neutralizing antibody (nAb) and seroresponse against Omicron BA.1, superiority of the nAb response against Omicron-BA.1, and non-inferiority of the nAb response against ancestral SARS-CoV-2 for second boosters of mRNA-1273.214 versus mRNA-1273 at days 29 and 91. The key secondary endpoint was the seroresponse difference of mRNA-1273.214 versus mRNA-1273 against ancestral SARS-CoV-2 at days 29 and day 91. Participants were sequentially enrolled and dosed with 50-µg of mRNA-1273 (*n* = 376) or mRNA-1273.214 (*n* = 437) as second booster doses. Here we present day 91 post-booster results. In participants with no pre-booster, severe acute respiratory syndrome coronavirus 2-infection (SARS-CoV-2), mRNA-1273.214 elicited Omicron-BA.1-nAb titers (95% confidence interval [CI]) that were significantly higher (964.4 [834.4-1114.7]) than those of mRNA-1273 (624.2 [533.1-730.9]) and similar to those of mRNA-1273 against ancestral SARS-CoV-2 at day 91. mRNA-1273.214 also induced higher binding antibody responses against Omicron BA.1 and alpha, gamma and delta variants than mRNA-1273. Safety profiles were similar for both vaccines. The Omicron-BA.1 bivalent vaccine improved antibody responses compared to mRNA-1273 through 90 days post-booster.

## Abstract (web summary)

Authors study antibody responses elicited against Omicron BA.1 and variants, by Omicron BA.1-bivalent mRNA-1273.214 vaccine compared with mRNA-1273 vaccination, when administered as a second booster, 90 days post-administration.

## Introduction

Booster immunization improves immune responses against severe acute respiratory syndrome coronavirus 2 (SARS-CoV-2) variants and vaccine effectiveness against coronavirus disease 2019 (COVID-19)^1–3^. The emergence of antigenically-divergent Omicron variants, required updated strategies for booster immunization^4–6^. As such, Omicron-containing, bivalent boosters are currently available in multiple geographies to address COVID-19 caused by Omicron variants^7,8^. The Omicron-BA.1-containing bivalent booster (mRNA-1273.214) demonstrated acceptable safety and higher neutralizing antibody responses against BA.1 and other variants compared to the original booster mRNA-1273 and remains widely used around the world^9,10^. An Omicron BA.4/BA.5 bivalent booster (mRNA-1273.222) was authorized in the US and elsewhere for the fall 2022 immunization campaign^11–14^.

We previously reported the day 29 interim results of an ongoing phase 2/3 study (NCT04927065) that evaluates the safety and immunogenicity of the Omicron-BA.1-containing bivalent booster mRNA-1273.214 administered as a second booster dose to participants who have previously received the original mRNA-1273 vaccine^9^. The 50-µg mRNA-1273.214 booster elicited higher neutralizing antibody (nAb) responses against Omicron BA.1, compared to the 50-µg mRNA-1273 booster, and exhibited a cross-neutralization ability against multiple Omicron variants including BA.4/BA.5 and BA.2.75 with a safety profile similar to that of mRNA-1273^9,15^. Although increased potency and breadth of the antibody response are highly desirable, it is also important to evaluate antibody persistence.

In this work, we present day 91 mRNA-1273.214 immunogenicity and safety data to address the question of antibody durability and longer-term safety with bivalent boosters. The day 91 immunogenicity objectives were pre-specified to evaluate whether the bivalent booster can induce superior responses against Omicron BA.1 compared to mRNA-1273. Results show that the booster elicits nAb responses that were superior against Omicron BA.1 and non-inferior against ancestral SARS-CoV-2 (D614G) compared to mRNA-1273 at 3 months after the booster dose, with a similar safety profile. Additionally, mRNA-1273.214 exhibits cross-neutralization against divergent variants not contained in the vaccine.

## Results

Between February 18th-March 8th, 2022 (part F, cohort 2) and March 8th-March 23rd, 2022 (part G), 819 participants were enrolled who had previously received the primary series of 100-µg mRNA-1273 and a first booster dose of 50-µg mRNA-1273, ≥3 months prior (Fig. S1 and Supplementary Methods) in the COVE trial (*n* = 461) or under US emergency use authorization (*n* = 358)^9^. Participants were enrolled in a sequential, non-randomized manner and received single second boosters of 50-µg mRNA-1273 (*n* = 376) or 50-µg bivalent mRNA-1273.214 (*n* = 437). The demographics and baseline characteristics of the participants were balanced in the 50-µg mRNA-1273.214 and 50-µg mRNA-1273 groups (Table S1), including age, race, and ethnicity. Median interval days (interquartile range [IQR)]) between second doses of mRNA-1273 in the primary series and the first booster of mRNA-1273 (245 [224–275] and 242 [225–260]), and between the first booster dose of mRNA-1273 and the second booster doses (136 [118–150] and 134 [118–150]) were similar between the mRNA-1273.214 and mRNA-1273 groups, respectively. Additionally, similar percentages of participants had evidence of prior SARS-CoV-2 infection in the mRNA-1273.214 (22%) and mRNA-1273 (27%) groups.

Median durations of follow-up days (IQR) were 113 (111–115) for the mRNA-1273.214 and 127 (125–132) for the mRNA-1273 boosters. The occurrences of solicited adverse reactions (ARs) within 7 days and the incidence of unsolicited adverse events (AEs) up to 28 days following mRNA-1273.214 were overall similar to those of mRNA-1273 as previously reported (Tables S2 and S3)^9^. In this interim analysis with longer-term follow-up than previously reported, the incidences of unsolicited AEs reported throughout to the cutoff date regardless of relationship to study vaccine (47.8% and 52.1%) and those considered related to study vaccination by the investigator (4.8% and 5.6%) were similar between the mRNA-1273.214 and the mRNA-1273 groups, respectively (Table S4). One (0.3%) death (fatal hypotension in a 73-year-old female with pre-existing vascular conditions who had an elective cardiac catheterization complicated by hypotension) occurred 64 days after mRNA-1273 immunization and was considered by the investigator to not be related to study vaccination. No events of myocarditis or pericarditis were reported and a total of 8 (1.8%) serious AEs occurred in the mRNA-1273.214 and 10 (2.7%) in the mRNA-1273 groups, all of which were considered unrelated to study vaccination by investigators (Table S5).

**Fig. 1:**
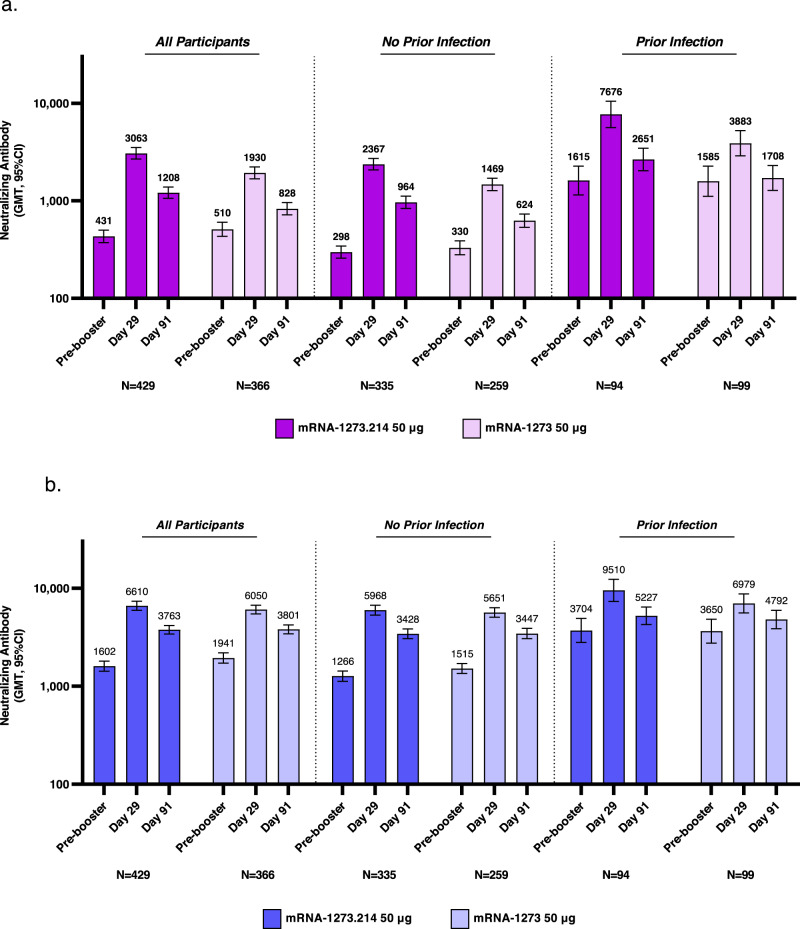
Observed neutralizing antibody titer against ancestral SARS-CoV-2 (D614G) and Omicron BA.1 variant after 50 µg of mRNA-1273.214 and mRNA-1273 administered as second booster. Unadjusted, observed pseudovirus neutralizing geometric mean titers (GMT [95% CI]) are provided for all participants regardless of prior SARS-CoV-2 infection pre-booster and those with and without SARS-CoV-2 infection, against the Omicron BA.1 variant (**a**) and ancestral SARS-CoV-2 (D614G) (**b**). Data are from participants with non-missing data at the time point. Eight participants in the mRNA-1273 50-µg group were missing pre-booster SARS-CoV-2 status. Antibody values reported as below the lower limit of quantification ([LLOQ] 18.5 for ancestral SARS-CoV-2 [D614G]; 19.9 for omicron BA. 1) were replaced by 0.5 × LLOQ. Values greater than the upper limit of quantification ([ULOQ] 45,118 for ancestral SARS-CoV-2 [D614G]; 15502.7 for Omicron BA.1) were replaced by the ULOQ if actual values are not available. 95% CIs were calculated based on the *t*-distribution of the log-transformed values then back transformed to the original scale for presentation. Observed nAb GMTs are summarized in Table S6.

Immunogenicity was evaluated in the per-protocol immunogenicity set (PPIS) of participants who received the planned booster doses and had pre-booster and day 29 antibody data available with no major protocol deviations. The analysis of the primary immunogenicity objectives was performed per the pre-specified testing strategy (Fig. S2) in participants without evidence of pre-booster SARS-CoV-2 infection (PPIS-negative). Immunogenicity was also assessed in the per-protocol immunogenicity set regardless of pre-booster infection status (PPIS) and in those with evidence of prior SARS-CoV-2 infection (Fig. S3). Previously published results included only day 29 antibody responses^9^. Unadjusted, observed nAb geometric mean titers (GMTs) 95% confidence intervals (95% CI) at day 29 post-boost in the PPIS-negative set against Omicron BA.1 were higher after the mRNA-1273.214 (2366.6 [2066.2–2710.7]) than mRNA-1273 (1468.7 [1266.2–1703.6]) booster, and similar against ancestral SARS-CoV-2 (D614G) (5968.1 [5315.4–6700.9]) and (5651.4 [5055.7–6317.3]) following the boosters, respectively (Table 1 and Figs. 1, S4, and S5). Model-estimated GMTs (95% CI) after adjusting for age groups and pre-booster titers at day 29 against Omicron BA.1 were 2469.7 (2255.5–2704.3) and 1419.1 (1280.8–1572.3) and against ancestral SARS-CoV-2 (D614G) were 6406.1 (5975.5–6867.8) and 5291.1 (4890.5–5724.5) after the mRNA-1273.214 and mRNA-1273 boosters, respectively. At day 91, observed GMTs (95% CI) against Omicron BA.1 were also higher after the mRNA-1273.214 (964.4 [834.4–1114.7]) than mRNA-1273 (624.2 [533.1–730.9]) booster and those against ancestral SARS-CoV-2 (D614G) were similar (3428.3 [3062.7–3837.6] and 3447.1 [3054.7–3889.9]) for the boosters. Estimated GMT (95% CI) against Omicron BA.1 were 997.5 (898.4–1107.4) and 602.7 (534.7–679.4) and against ancestral SARS-CoV-2 (D614G) were 3595.6 (3334.8–3876.8) and 3257.3 (2986.3–3552.9) for mRNA1273.214 and mRNA-1273, respectively. The geometric mean ratios (GMRs [97.5% CI]) for the mRNA-1273.214 versus mRNA-1273 GMTs against Omicron BA.1 at both days 29 (1.74 [1.49–2.04]) and 91 (1.66 [1.38–1.99]) met the pre-specified criterion (lower bound of the 97.5% CI of GMR > 1) for superiority, and the GMRs against ancestral SARS-CoV-2 (D614G) at both days 29 (1.21 [1.07–1.37]) and 91 (1.10 [0.97–1.26]), met the pre-specified criterion for non-inferiority (lower bound of 97.5% CI of GMR ≥ 0.67).

**Table 1:** Immunogenicity analysis of ancestral SARS-CoV-2 (D614G) and Omicron BA.1 after 50 µg of mRNA-1273.214 and mRNA-1273 administered as second booster doses in participants with no prior SARS-CoV-2 infection

	Ancestral SARS-CoV-2 (D614G)		Omicron BA.1	
	50 µg mRNA-1273.214 booster dose	50 µg mRNA-1273 booster dose	50 µg mRNA-1273.214 booster dose	50 µg mRNA-1273 booster dose
	*N* = 335	*N* = 259	*N* = 335	*N* = 259
Pre-booster, *n*^a^	335	259	335	259
Observed GMT (95% CI)^b^	1265.9 (1119.9 to 1431.0)	1515.4 (1347.5 to 1704.2)	297.6 (258.4 to 342.8)	329.5 (280.0 to 387.9)
Day 29, *n*^a^	335	259	335	259
Observed GMT (95% CI)^b^	5968.1 (5315.4 to 6700.9)	5651.4 (5055.7 to 6317.3)	2366.6 (2066.2 to 2710.7)	1468.7 (1266.2 to 1703.6)
GMFR (95% CI)^b^	4.7 (4.4 to 5.1)	3.7 (3.4 to 4.1)	8.0 (7.2 to 8.8)	4.5 (4.0 to 5.0)
Estimated GMT (95% CI)^c^	6406.1 (5975.5 to 6867.8)	5291.1 (4890.5 to 5724.5)	2469.7 (2255.5 to 2704.3)	1419.1 (1280.8 to 1572.3)
GMR (97.5% CI)^c^	1.21 (1.07 to 1.37)		1.74 (1.49 to 2.04)^g^	
Day 91, *n*^a^	328	243	324	243
Observed GMT (95% CI)^b^	3428.3 (3062.7 to 3837.6)	3447.1 (3054.7 to 3889.9)	964.4 (834.4 to 1114.7)	624.2 (533.1 to 730.9)
GMFR (95% CI)^b^	2.7 (2.5 to 2.9)	2.3 (2.2 to 2.5)	3.2 (2.8 to 3.6)	1.9 (1.7 to 2.1)
Estimated GMT (95% CI)^c^	3595.6 (3334.8 to 3876.8)	3257.3 (2986.3 to 3552.9)	997.5 (898.4 to 1107.4)	602.7 (534.7 to 679.4)
GMR (97.5% CI)^c^	1.10 (0.97 to 1.26)		1.66 (1.38 to 1.99)^g^	
Day 29 SRR, *n*/N1 %^d^	335/335, 100	259/259, 100	334/334, 100	255/257, 99.2
(95% CI)^e^	(98.9 to 100)	(98.6 to 100)	(98.9 to 100)	(97.2 to 99.9)
Difference, % (97.5% CI)^f^	0		1.5 (−1.1 to 4.1)	
Day 91 SRR, *n*/N1 %^d^	328/328, 100	242/243, 99.6	318/323, 98.5	232/241, 96.3
(95% CI)^e^	(98.9 to 100.0)	(97.7 to 100.0)	(96.4 to 99.5)	(93.0 to 98.3)
Difference, % (97.5% CI)^f^	0.9 (−1.6 to 3.5)		2.1 (−1.6 to 5.8)	

Unadjusted observed antibody values assessed by pseudovirus neutralizing antibody assay reported as below the LLOQ (18.5 for ancestral [D614G] and 19.9 for Omicron BA.1) are replaced by 0.5 × LLOQ. Values greater than ULOQ (45,118 for ancestral SARS-CoV-2 [D614G] and 15,502.7) for Omicron BA.1 are replaced by the ULOQ if actual values are not available. Includes participants in the per-protocol immunogenicity set without evidence of pre-booster SARS-CoV-2 infection (PPIS-negative). Participant immune response data is censored at the last date of study participation (study discontinuation, study completion, or death), non-study COVID-19 vaccination date, or data cutoff/extraction date, whichever is the earliest.

*ANCOVA* analysis of covariance, *CI* confidence interval, *GMT* geometric mean titer, *GMFR* geometric mean fold rise (days 29 and 91 post-baseline timepoint over pre-booster baseline), *GMR* geometric mean ratio, mRNA-1273.214 versus mRNA-1273, *LLOQ* lower limit of quantification, *LS* least squares, *SRR* seroresponse rate, *ULOQ* upper limit of quantification.

^a^Number of participants with non-missing data at the timepoint (baseline or post-baseline). Estimated GMTs from an ANCOVA model, adjusted for covariates were used for assessments of differences in antibody responses (GMRs, SRRs).

^b^95% CI based on the t-distribution of log-transformed values or difference in the log-transformed values for GMT value and GMFR, respectively, then back transformed to the original scale.

^c^Log-transformed antibody levels are analyzed using an ANCOVA model with the treatment variable as fixed effect, adjusting for age group (<65, ≥65 years) and pre-booster titers. The resulting LS means, difference of LS means, and 95% CI and 97.5% CI are back transformed to the original scale.

^d^Seroresponse at a participant level based on pre-injection 1 baseline, defined as a change from <LLOQ to ≥4 × LLOQ, or at least a fourfold rise if baseline is ≥LLOQ; comparison to pre-vaccination baseline for participants without pre-injection 1 antibody titer information who had a corresponding day 29 post-boost assessment and negative SARS-CoV-2 status at pre-injection 1 of the primary series, seroresponse was defined as ≥4 x LLOQ and antibody titers were imputed as <LLOQ at pre-injection 1 of the primary series. For participants who were without SARS-CoV-2 status information at pre-injection 1 of primary series, their pre-booster SARS-CoV-2 status was used to impute their SARS-CoV-2 status at their pre-injection 1 of the primary series. Percentages were based on the number of participants with non-missing data at baseline and the corresponding time point.

^e^95% CI is calculated using the Clopper–Pearson method.

^f^97.5% CI was calculated by stratified Miettinen–Nurminen method adjusted by age group. The stratified Miettinen–Nurminen estimate and the CI cannot be calculated when the seroresponse rate in both groups is 100%, absolute difference is reported.

^g^Exceeded non-inferiority criteria and met superiority criteria including lower bound CI > 1 and testing sequence.

Seroresponse rates (SRR [95% CI]) at day 29 were 100% (98.9–100%) and 99.2% (97.2–99.9%) against Omicron BA.1 and 100% (98.9–100% and 98.6–100%) against ancestral SARS-CoV-2 (D614G) for the mRNA-1273.214 and mRNA-1273 boosters, respectively, and at day 91, SRRs were 98.5% (96.4–99.5%) and 96.3% (97.2–99.9%) against Omicron BA.1 and 100% (98.9–100%) and 99.6% (97.7–100.0%) against ancestral SARS-CoV-2 (D614G). The estimated SRR (97.5% CI) differences between mRNA-1273.214 and mRNA-1273 at days 29 (1.5% [−1.1 to 4.1%] and 0) and 91 (2.1% [−1.6 to 5.8%] and 0.9% [−1.6 to 3.5%]) against Omicron BA.1 and ancestral SARS-CoV-2 (D614G), respectively, both met the prespecified non-inferiority criterion (lower bound of the 97.5% CI > −10%).

Results were consistent in all participants regardless of prior SARS-CoV-2 infection (PPIS) and the PPIS participants who had evidence of pre-booster SARS-CoV-2 infection, with higher nAb titers observed at days 29 and 91 against Omicron BA.1 for the mRNA-1273.214 versus mRNA-1273 booster and similar titers against ancestral SARS-CoV-2 (D614G) at both days for the two boosters (Figs. 1, S4, and S5 and Table S6). Neutralizing titers against Omicron BA.1 were also consistently higher with mRNA-1273.214 than mRNA-1273 at days 29 and 91 among those ≥65 years and 18–<65 years of age in the PPIS-negative group (Fig. S6 and Table S7). The GMTs against ancestral SARS-CoV-2 (D614G) were similar for the two boosters at days 29 and 91 among those ≥65 years and 18–<65 years, respectively, in the PPIS-negative set.

In participants in the PPIS set with no prior SARS-CoV-2 infection, binding antibody (bAb) GM-levels were higher for mRNA-1273.214 than mRNA-1273 against both Omicron BA.1 and ancestral SARS-CoV-2, and alpha, gamma, and delta variants at both days 29 and 91 (Fig. S7 and Tables S8 and S9). Across variants, the GMRs of bAb levels for mRNA-1273.214 versus mRNA-1273 ranged from 1.10 (1.03–1.19) to 1.24 (1.15–1.34) at day 29 and 1.20 (1.11–1.30) to 1.26 (1.16–1.37) at day 91. Seroresponse rates of 100% were observed for all variants and the differences were 0%.

In an exploratory analysis of subsets of participants, antibody responses against omicron BA.4/BA.5, BQ.1.1 and XBB.1 variants also increased following the mRNA-1273.214 booster at day 29 (Supplemental Methods, Fig. S8 and Table S10), although the titers were lower than those against the matched BA.1. variant. The nAb GMTs rose 3.6–5.0-fold in participants without prior SARS-CoV-2 infection (*n* = 40) and 2.9–3.2-fold in those with prior SARS-CoV-2 infection (*n* = 20) against Omicron BA.4/BA.5, BQ.1.1 and XBB.1, respectively and 6.8 and 4.5-fold for Omicron BA.1.

As of the data cutoff date (July 6, 2022), among all participants regardless of pre-booster SARS-CoV-2 infection status, the incidences of SARS-CoV-2 infection and COVID-19 events starting 14 days post-booster were balanced between the mRNA-1273.214 and mRNA-1273 groups (Table S11). There were 61 (14.0%) and 48 (12.8%) SARS-CoV-2 infections, 19 (4.3%) and 18 (4.8%) asymptomatic infections, and 39 (8.9%) and 26 (6.9%) COVID-19 events per the COVE trial definition, and 42 (9.6%) and 30 (8.0%) COVID-19 events per the CDC definition in the mRNA-1273.214 and mRNA-1273 groups, respectively. The incidences of infections were similar in the per-protocol efficacy set of participants with no prior SARS-CoV-2 infection. No emergency room visits, or hospitalizations attributed to COVID-19 were seen.

## Discussion

The Omicron BA.1-containing bivalent booster vaccine mRNA-1273.214 consistently induced higher neutralizing antibody responses against the Omicron BA.1 variant compared to the original mRNA-1273 vaccine. The mRNA-1273.214 booster had a safety profile simlar to that of the original vaccine through approximately 3.5 months after the bivalent booster dose. Neutralizing antibody titers against Omicron BA.1 were significantly higher 90 days after the booster dose with the bivalent than the mRNA-1273 vaccine, with no decrement in the response against ancestral SARS-CoV-2 (D614G). The binding antibody data also indicate improved antibody persistence with the bivalent booster against multiple variants. The higher antibody titers at days 29 and 91 with the bivalent booster could, at least in part, be due to new, variant-specific germinal centers induced after immunization with variant-targeting boosters^16^. Overall, the results were generally consistent regardless of age and pre-booster SARS-CoV-2 infection, although nAb titers were higher and increases in nAb titers from baseline were lower among those with prior infection. Given the large proportion of the US population that have been previously infected with SARS-CoV-2 and a higher risk of severe disease in older individuals, these findings indicate that boosters will benefit these groups^17^. Additionally, mRNA-1273.214 exhibited cross-neutralization against divergent variants not contained in the vaccine including Omicron BA.4/BA.5, BQ.1.1 and XBB.1, although the antibody titers for these variants were lower compared to the matched Omicron BA.1 variant. These results are overall consistent with a randomized and active-controlled clinical trial of the mRNA-1273.214 booster versus mRNA-1273 which suggested a trend towards improved relative vaccine efficacy against variants antigenically closer to the variant contained in the vaccine^18^.

Limitations of the study include that it was not randomized and although a sequential design was used, baseline characteristics, including intervals between prior doses, were balanced between the two groups. The interpretation of results in the study is based upon immunogenicity data and the biological relevance of the higher antibody responses elicited by the mRNA-1273.214 booster against Omicron BA.1 compared with those of mRNA-1273, has yet to be determined. We also do not have information on the variants that caused prior SARS-CoV-2 infections nor the timing of those infections in relation to boosting. Although the incidence of infections post-booster is provided as an exploratory analysis, the study was not designed to evaluate vaccine efficacy and post-booster effectiveness will need to be evaluated in observational studies. Only humoral responses were assessed and cellular responses warrant characterization in ongoing studies^16^.

In conclusion, the bivalent Omicron-BA.1 containing mRNA-1273.214 elicited higher nAb responses against Omicron BA.1 and other variants compared to mRNA-1273 when administered as a second booster through 90 days post-booster in the absence of evident safety concerns. Although enhanced antibody responses have the potential to confer improved protection against COVID-19, real-world vaccine effectiveness studies are needed to address this question.

## Methods

### Study design and participants

This is an open-label, ongoing phase 2/3 study (NCT04927065) which evaluates the immunogenicity, safety, and reactogenicity of bivalent booster vaccine mRNA-1273.214 compared to the currently-authorized mRNA-1273 booster vaccine in adults who had previously received 2-dose primary series (100 µg) and first booster doses (50 µg) of mRNA-1273 in COVE^19,20^ or under US emergency use authorization, enrolled in a sequential, non-randomized manner. Participants received single second boosters of 50-µg mRNA-1273 (part F, cohort 2) or 50-µg bivalent mRNA-1273.214 (part G). Enrollment of the mRNA-1273.214 50-µg second boost arm was initiated upon completion of enrollment of the mRNA-1273 50-µg arm in cohort 2 of part F. The 50-µg mRNA-1273 booster serves as a non-contemporaneous within-study comparator. Part G interim results at day 29 were previously described^9^ and day 91 results are reported here.

The trial is being conducted across 23 US sites, in accordance with the International Council for Harmonisation of Technical Requirements for Registration of Pharmaceuticals for Human Use, Good Clinical Practice guidelines. The central Institutional Review Board/Ethics Committee (Advarra, Inc., 6100 Merriweather Drive, Columbia, MD 21044) approved the protocol and consent forms. All participants provided written informed consent.

Eligible participants included healthy male and female adults >18 years of age. Persons with known histories of SARS-CoV-2 infection ≤3 months from screening or significant exposure to SARS-CoV-2 or COVID-19 14 days prior to screening were excluded (additional inclusion/exclusion criteria are provided in the Supplement).

### Trial vaccine

The bivalent mRNA-1273.214 50-µg vaccine contains two mRNAs (1:1, 25-µg each) encoding the prefusion-stabilized spike glycoproteins of ancestral SARS-CoV-2 (Wuhan-Hu-1) and the Omicron variant (B.1.1.529 [BA.1]). The monovalent mRNA-1273 50-µg vaccine contains a single mRNA encoding the spike glycoprotein of ancestral SARS-CoV-2 (Wuhan-Hu-1). The mRNA-1273.214 and mRNA-1273 boosters were administered intramuscularly at 50 µg in a 0.5 mL volume.

### Assessments

#### Safety

The primary safety objective was to evaluate the safety and reactogenicity of 50-µg mRNA‑1273.214 and 50-µg mRNA-1273 when administered as second booster doses. Safety assessments included solicited local and systemic adverse reactions within 7 days and unsolicited AEs within 28 days post-booster administration. Serious AEs, AEs leading to discontinuation from study vaccine and/or participation, medically attended AEs, and AEs of special interest are being assessed from day 1 through the entire study period (~12 months).

### Immunogenicity

The pre-specified primary immunogenicity objectives were to demonstrate non-inferiority of neutralizing antibody (nAb) responses based on the geometric mean titer (GMT) ratio (GMR) and seroresponse (SRR) difference, superiority of nAb responses based on GMR against Omicron BA.1 and non-inferiority of the nAb responses based on GMR against ancestral SARS-CoV-2 (D614G), 28 days (day 29) or 90 days (day 91) after second boosters of mRNA 1273.214 (50 µg) compared with mRNA-1273 (50 µg). The pre-specified key secondary objective was to demonstrate non-inferiority (seroresponse rate difference) against ancestral SARS-CoV-2 (D614G) at day 29 or 91 after second booster of mRNA-1273.214 50 µg compared with mRNA-1273 50 µg.

Neutralizing antibody GMTs were assessed at inhibitory dilutions 50% (ID50) using fully validated SARS-CoV-2 spike-pseudotyped lentivirus neutralization assays against pseudoviruses containing the SARS-CoV-2 full-length spike proteins of ancestral SARS-CoV-2 (D614G) or Omicron BA.1 variant^21^. Geometric mean (GM)-levels of spike-binding antibody (bAb) were also assessed using a Meso Scale Discovery [MSD] assay against ancestral SARS-CoV-2, gamma (P.1), alpha (B.1.1.7), delta [B.1.617.2; AY.4], and Omicron (BA.1) variants. Immunogenicity assays are further described in the supplement.

### Incidence of SARS-CoV-2 infections

An exploratory objective of the study is to assess symptomatic and asymptomatic SARS-CoV-2 infection. SARS-CoV-2 infection comprises a combination of symptomatic infection (COVID-19) and asymptomatic SARS-CoV-2 infection for participants who had negative SARS-CoV-2 status pre-booster. Symptomatic infection was evaluated using the primary case definition in the COVE study^19,20^ and using a secondary case definition based on the Centers for Disease Control and Prevention (CDC) criteria^22^. Asymptomatic SARS-CoV-2 infection was defined as having a positive reverse-transcriptase polymerase chain reaction (RT-PCR) test or a positive serologic test for anti-nucleocapsid antibody (Elecsys, Roche) after a negative test at the time of enrollment, in the absence of symptoms.

### Statistical analysis

Safety was evaluated in the safety set consisting of all participants who received second boosters and solicited ARs were evaluated in the solicited safety set. The per-protocol immunogenicity set (PPIS) consists of all participants in the full analysis set who received the planned booster doses, had pre-booster and day 29 antibody data against Omicron BA.1 available with no major protocol deviations. Primary immunogenicity objectives were assessed in the PPIS–SARS-CoV-2-negative set (primary analysis set) comprised of participants with no evidence of SARS-CoV-2 infection defined as having both negative virologic (RT-PCR for SARS-CoV-2 infection) and serologic (bAb against SARS-CoV-2 nucleocapsid) tests. Analyses were also performed in all participants in the PPIS regardless of SARS-CoV-2 infection pre-booster status, and in those who had evidence of prior SARS-CoV-2 infection pre-booster defined as having positive tests for binding antibody against SARS-CoV-2 nucleocapsid or RT-PCR of SARS-CoV-2 infection at baseline. Participants with SARS-CoV-2 infection were excluded from the primary analysis.

The primary immunogenicity objectives were tested using a pre-specified hierarchical approach hypotheses testing strategy (Fig. S2, Supplemental Methods and online Statistical Analysis Plan). There were 8 specified hypotheses of (4 identical hypotheses each at days 29 and 91) to be evaluated at days 29 and 91. Two interim analyses were planned at days 29 and 91 with a two-sided alpha (0.025) allocated at each time point to preserve the family-wise type I error rate (0.05 two-sided) for immunogenicity hypothesis testing. The day 29 interim data were reported previously, and both day 29 and 91 data are summarized in this report^9^. The superiority of the nAb response against Omicron BA.1 after a second booster dose of 50-µg mRNA‑1273.214 compared with 50-µg mRNA‑1273 was tested only after meeting non-inferiority criteria for the three primary objectives^23^: antibody response against Omicron BA.1 after the second booster doses of 50-µg mRNA‑1273.214 versus 50-µg mRNA‑1273 based on GMR, antibody response against Omicron BA.1 after the second booster doses of 50-µg mRNA‑1273.214 versus 50-µg mRNA‑1273 based on the difference in response rate, and antibody response against ancestral SARS-CoV-2 (D614G) after the second booster doses of 50-µg mRNA‑1273.214 versus 50-µg mRNA‑1273 based on GMR. All tests were based on an alpha of 0.025 (two-sided) at day 29 and 91. If the primary objectives were met, the key secondary objective of non-inferiority of the nAb response after second booster doses of 50-µg mRNA-1273.214 compared to 50-µg mRNA-1273 against ancestral SARS-CoV-2 (D614G) based on the SRR-difference was then tested (alpha=0.025, two-sided). In the study, non-inferiority is considered met when the lower bound of the 97.5% confidence interval (CI) of the GMR is ≥0.67 and the seroresponse rate-difference is >−10%. Superiority is considered met when the lower bound of the 97.5% CI of the GMR is >1^12,24^. If all primary and key secondary objectives were met at day 29, the hypotheses at day 91 could be tested at alpha of 0.05 (2-sided).

Unadjusted, observed GMTs (95% CI) using t-distribution of log-transformed antibody titers are presented. Additionally, for the assessment of differences in antibody responses between the mRNA-1273.214 and mRNA-1273 groups, GMTs were estimated using an analysis of covariance (ANCOVA) model, with post-booster antibody titers as the dependent variable and the group variable of mRNA-1273.214 and mRNA-1273 as the fixed effect, adjusted for age groups (<65, ≥65 years) and pre-booster antibody titers. Adjusted GMTs (95% CI) were estimated by the geometric least square mean from the model and differences in antibody responses (GMR) between groups estimated by the ratio of geometric least mean square (97.5% CIs) are provided (as day 91 results passed all tests at both the 0.05 and 0.025 level, 97.5% CI are presented). Seroresponses defined as a change from <lower limit of quantification [LLOQ] to ≥4 × LLOQ, or at least a 4-fold rise if the baseline is ≥LLOQ with 95% CI (Clopper–Pearson) and response rate differences between mRNA-1273.214 and mRNA-1273 groups (97.5% CI; Miettinen–Nurminen), adjusting for age groups are provided. For the primary and key secondary objectives, 97.5% CIs are provided.

Additional analyses included assessment of the primary immunogenicity endpoints in the PPIS, an analysis of participants with prior evidence of SARS-CoV-2 infection and an analysis that excluded antibody data of participants who acquired SARS-CoV-2 infection during study. These analyses were performed using an ANCOVA model (Supplementary Methods). The observed bAb GM-levels against variants and bAb differences between mRNA-1273.214 and mRNA-1273 groups based on GMRs (95% CIs) assessed by ANCOVA are also provided. An analysis of observed GMTs against the Omicron BQ.1.1 and XBB.1 variants was also performed in random subsets of recipients (*n* = 60; 40 without prior infection and 20 with prior infection) in the mRNA-1273.214 group (Supplementary Methods).

The number and percentage of participants with asymptomatic or symptomatic SARS-CoV-2 infection and COVID events are summarized. All analyses were conducted using SAS Version 9.4 or higher.

### Reporting summary

Further information on research design is available in the Nature Portfolio Reporting Summary linked to this article.

### Supplementary information

Supplementary information

Reporting Summary

### Source data

Source Data

## Data Availability

Data associated with this study are provided in the paper or supplementary materials. As the trial is ongoing, access to patient-level data and supporting clinical documents by qualified external researchers may be available upon request and subject to review once the trial is complete. The protocol and statistical analysis plan are provided as supplementary materials. Such requests can be made to Dr. Spyros Chalkias, Moderna Inc., 200 Technology Square, Cambridge, MA 02139, USA. Source data are provided with this paper.
